# Sustained Cognitive Improvement in Alzheimer’s Disease Patients Following a Precision Medicine Protocol: Case Series

**DOI:** 10.3390/biomedicines12081776

**Published:** 2024-08-06

**Authors:** Dale E. Bredesen, Mary Kay Ross, Stephen Ross

**Affiliations:** 1 Precision Brain Health Program, Pacific Brain Health Center, Pacific Neuroscience Institute, Santa Monica, CA 90404, USA; 2 Institute for Personalized Medicine, Savannah, GA 31406, USA; mkrossmd@gmail.com (M.K.R.); stephen.ross@brainhealthandresearch.com (S.R.)

**Keywords:** mild cognitive impairment, treatment, donepezil, anti-amyloid therapy, personalized medicine

## Abstract

Arguably, the most important parameter in treating cognitive decline associated with Alzheimer’s disease is the length of time in which improvement, if achieved at all, is sustained. However, monotherapies such as donepezil and memantine are associated with a more rapid decline than no treatment in patients over multi-year follow-ups. Furthermore, anti-amyloid antibody treatment, which at best simply slows decline, is associated with accelerated cerebral atrophy, resulting in earlier dementia-associated brain volumes for those treated at the MCI stage than untreated patients. In contrast, a precision medicine approach, in which the multiple potential drivers of cognitive decline are identified for each patient and then targeted with a personalized protocol (such as ReCODE), has led to documented improvements in patients with cognitive decline, but long-term follow-up (>5 years) has not been reported previously. Therefore, here, we report sustained cognitive improvement, in some cases for over a decade, in patients treated with a precision medicine protocol—something that has not been reported in patients treated with anti-cholinesterase, glutamate receptor inhibitory, anti-amyloid, or other therapeutic methods. These case studies warrant long-term cohort studies to determine how frequently such sustained cognitive improvements occur in patients treated with precision medicine protocols.

## 1. Introduction

Most clinical trials of candidate therapeutics for Alzheimer’s disease report outcomes after relatively short or intermediate treatment intervals such as 12 or 18 months, but do not include follow-up after 5–10 years or more. This is particularly important because of the reviews suggesting that the major current treatments, such as donepezil, memantine, and anti-amyloid antibodies, are associated not just with lack of sustained improvement, but unfortunately with long-term outcomes that are worse than the outcomes associated with no treatment [1,2]. One of the implications of these findings is that these standard treatment modalities are somehow failing to address the underlying cause or contributors to the cognitive decline associated with Alzheimer’s disease (AD), or are indeed addressing the underlying cause or contributors but without sufficient efficacy or sustained effects.

Beginning in 2014, reports of the reversal of cognitive decline in patients with AD—at the stages of subjective cognitive impairment, mild cognitive impairment, and dementia—began to appear [3,4,5,6], based on an earlier theoretical publication [7]. These reports included case studies [3,6,8], a pragmatic cohort study [9], and proof-of-concept clinical trials [4,5], with a randomized, controlled trial ongoing (https://www.dementiareversaltrial.com/; https://classic.clinicaltrials.gov/ct2/show/NCT05894954) (accessed on 20 May 2024). Improvements were documented in cognitive tests such as the Montreal Cognitive Assessment (MoCA) and an on-line cognitive assessment provided by CNS Vital Signs [10], partner assessments based on the Alzheimer’s Questionnaire (AQ-21) [11], brain training scores, metabolic profiles, and MRI volumetrics.

It has been observed by some of the treating physicians, patients, and the patients’ family members that some of the patients reported in these publications have now sustained their improvement for years, but to date, there have not been published reports of these long-term, sustained improvements. Therefore, here, we report long-term follow-up of seven patients who had documented cognitive improvement that has now been sustained for up to 11 years. These outcomes cannot be compared directly to those arising from clinical trials, since these are anecdotal; however, their documentation suggests that a long-term trial to determine how frequently such outcomes occur is warranted.

## 2. Detailed Case Description

These seven case studies were taken from patients evaluated and treated by the authors. No identifying information was included. The patients consented to the descriptions. The evaluation and treatment methods have been described previously [12].

### 2.1. Patient 1

This 75-year-old female professor was described previously [3], but at that time, she had only 18 months of follow-up. She has now been treated for seven years. She presented with paraphasic errors, severe enough that her speech was unintelligible to some of her listeners. She also developed depression, for which she was prescribed an antidepressant. She began to struggle with spelling, shopping, cooking, and working at the computer, as well as completing a gingerbread man for her granddaughter, despite having done this without difficulty many times previously. Her symptoms progressed, and she began to forget daily tasks, becoming very concerned when forgetting to pick up her grandchildren at school twice in two weeks.

She was found to be heterozygous for the ε4 allele of apolipoprotein E (ApoE 3/4). An amyloid PET scan (florbetapir) was positive. MRI revealed a hippocampal volume at the 14th percentile for her age. High-sensitivity C-reactive protein (hs-CRP) was 1.1 mg/L, fasting insulin 5.6 mIU/L, hemoglobin A1c 5.5%, homocysteine 8.4 micromolar, vitamin B12 471 pg/mL, free triiodothyronine (free T3) 2.57 pg/mL, thyroid-stimulating hormone (TSH) 0.21 mIU/L, albumin 3.7 g/dL, globulin 2.7 g/dL, total cholesterol 130 mg/dL, triglycerides 29 mg/dL, serum zinc 49 mcg/dL, complement factor 4a (C4a) 7990 ng/mL, transforming growth factor beta-1 (TGF-β1) 4460 pg/mL, and matrix metalloprotease-9 497 ng/mL.

Mild cognitive impairment (MCI) associated with AD was diagnosed, and she began a trial of an anti-amyloid antibody (solanezumab), but was not told whether she was in the treatment group or the control group. However, with each administration, her cognition became worse for 3–5 days, then returned gradually toward her previous MCI status. After she had become worse after each of the first several treatments, she discontinued her participation in the study.

She began treatment with the precision medicine approach described previously [4]. Her MoCA score increased from 24 to 30 over 17 months. Hippocampal volume increased from the 14th percentile to the 28th percentile for her age. Her symptoms resolved: her ability to spell returned, her speech improved, and her ability to shop, cook, and work at the computer all improved and remained stable on follow-up at 17 months.

Follow-up at seven years: She has sustained her improvement in all areas, with one period of secondary decline, and her granddaughter said, “It’s pretty crazy how much better” her memory has been. During years three to five on the protocol, she described her cognition as having been “the best in 20 years”. However, in her sixth year on the protocol, her cognition began to decline once again. She noticed that she had difficulty recalling names and often mixed up names, often forgot what she had planned to do when undertaking a task, often forgot appointments, lost the ability to do simple math problems, often felt overwhelmed, and often lost her train of thought in conversations.

Her scores declined on BrainHQ, a computer-based brain-training program that she uses daily. She was not able to match the scores she had attained the previous year. Furthermore, her score on a computer-based neuropsychological assessment, CNS Vital Signs, decreased from the 73rd percentile to the 61st percentile. Her MoCA score declined from 30 to 28. She developed depression and noted “brain fog”, with slower responses and more difficulty in problem solving. Based on her new symptoms and declining cognitive scores, she underwent further evaluation.

Severe obstructive sleep apnea was identified, with an apnea/hypopnea index (AHI) of 40. Nasal swabs revealed the fungus *Cryptococcus laurentii*. Urinary mycotoxin studies were positive for ochratoxin A, gliotoxin, and mycophenolic acid, and it was noted that her home had new water damage.

She began CPAP treatment for her sleep apnea. Fluconazole and an anti-fungal nasal spray were prescribed for her *Cryptococcus laurentii*. She began a detoxification protocol for her mycotoxin exposure, and her home was remediated.

Her symptoms once again resolved. Her depression lifted, her brain fog resolved, her problem solving improved, and her CNS Vital Signs neurocognitive index increased from the 61st percentile to the 86th percentile, which is higher than any of her previous scores. Her brain training scores also improved.

Comment: This patient had well-documented MCI in association with underlying AD pathology, with a positive amyloid PET scan, ApoE4 heterozygosity, and chronic cognitive decline, presenting with memory, language, spatial, and executive dysfunction. She responded to a personalized, multi-modal treatment approach, and sustained her improvement for six years, but then began to decline once again. Further evaluation revealed sleep apnea, nasal colonization with *Cryptococcus laurentii* (which is typically a skin pathogen rather than a rhinosinal pathogen), and mycotoxin exposure, and with treatment, her symptoms once again resolved.

### 2.2. Patient 2

This is a follow-up on a patient described in a previous publication [8]. A 69-year-old entrepreneur and professional man presented with 11 years of slow progressive memory loss, which had accelerated over the last one to two years. In 2002, at age 58, he had been unable to recall the combination of the lock on his locker. In 2003, he had an FDG PET scan, which was read as showing a pattern typical for early AD, with reduced glucose utilization in the parietotemporal cortices bilaterally and left > right temporal lobes, but preserved utilization in the frontal lobes, occipital cortices, and basal ganglia. In 2003, 2007, and 2013, he had quantitative neuropsychological testing, which showed a reduction in CVLT (California Verbal Learning Test), a Stroop color test at the 16th percentile, and auditory delayed memory at the 13th percentile. He was noted to be heterozygous for ApoE4 (3/4). He had progressive difficulty recognizing faces at work (prosopagnosia), and had to have his assistants prompt him with the daily schedule. He also recalled an event during which he was several chapters into a book before he finally realized that it was a book he had read previously. In addition, he lost an ability he had had for most of his life: the ability to add columns of multi-digit numbers rapidly in his head.

He was advised that, given his status as an AD patient and his progression, as well as his poor performance on the 2013 test, he should begin to “get his affairs in order”. His business was in the process of being shut down due to his inability to continue work.

His laboratory values included a homocysteine value of 18 µmol/L, CRP < 0.5 mg/L, 25-hydroxycholecalciferol 28 ng/mL, hemoglobin A1c 5.4%, serum zinc 78 mcg/dL, serum copper 120 mcg/dL, copper/zinc ratio of 1.54, ceruloplasmin 25 mg/dL, pregnenolone 6 ng/dL, testosterone 610 ng/dL, albumin/globulin ratio of 1.3, cholesterol 165 mg/dL (on atorvastatin), HDL 92 mg/dL, LDL 64 mg/dL, triglycerides 47 mg/dL, AM cortisol 14 mcg/dL, free T3 3.02 pg/mL, free T4 1.27 ng/L, TSH 0.58 mIU/L, and BMI 24.9.

He began on the personalized therapeutic program described in the original publication [8], and after six months, he, his wife, and co-workers all noted improvement. He lost 10 pounds. He was able to recognize faces at work once again, was able to remember his daily schedule once again, and was able to perform his work. He was also noted to be more rapid with his responses. His life-long ability to add columns of numbers rapidly in his head returned. His wife pointed out that, although he had clearly shown improvement, the more striking effect was that he had been accelerating in his decline over the prior year or two, and that had been halted.

After 22 months on the program, he returned for follow-up quantitative neuropsychological testing, which revealed marked improvement: CVLT-IIB had increased from the 3rd percentile to the 84th percentile, total recognized hits improved from <1st to the 50th percentile, CVLT-II improved from the 54th to the 96th percentile, auditory delayed memory improved from the 13th to the 79th percentile, reverse digit span improved from the 24th to the 74th percentile, and processing speed improved from the 93rd to the 98th percentile. His business was reinvigorated, and a new site was added to the previous sites of operation.

Follow-up at ten years: He has sustained his improvement, continued to work without difficulty, continued to operate his business at multiple sites, continued to be highly successful, enjoyed traveling, and not shown signs of declining.

Comment: This patient has well-documented AD, with an ApoE4-positive genotype, characteristic FDG-PET scan, characteristic abnormalities on neuropsychological testing, well-documented decline on longitudinal quantitative neuropsychological testing, and progression of symptoms. After two years on the protocol, his symptoms and neuropsychological testing improved markedly. The neuropsychologist who performed and evaluated his testing pointed out that his improvement was beyond that which had been observed in the neuropsychologist’s 30 years of practice. He has continued to follow the protocol and has sustained his improvement for ten years.

### 2.3. Patient 3

A 44-year-old man presented with cognitive decline of a four-year duration, starting with difficulty keeping up with his work schedule and forgetting his previous days’ activities. He also had difficulty with navigation, which impacted his job as a yacht captain, and noted some trouble with word finding and name recall. His wife was concerned that he may be developing early onset AD (EOAD), and noted that he had a very strong family history for AD.

He underwent laboratory testing and was found to be homozygous for the ApoE ε4 allele, with no mutations characteristic of familial AD (FAD) in the amyloid precursor protein (APP), presenilin-1 (PSEN1), or presenilin-2 (PSEN2) gene. He was also found to be heterozygous for the methylene tetrahydrofolate reductase (MTHFR) C677T mutation. His copper was 102 mcg/dL, zinc 75 mcg/dL, and homocysteine 24.5 µmol/L. His MMP-9 was elevated at 418 ng/mL, TGF-β1 was 3997 pg/mL, and he was found to be MARCoNS (multiple antibiotic resistance coagulase-negative Staph)-positive. His vitamin B12 was low at 317 pg/mL, vitamin D was 31 ng/mL, and his omega-3 fatty acid index was low at <2.0%. He was found to have elevated mercury levels, as determined by a pre- and post-heavy metal provocation test.

His initial evaluation included a MoCA score of 26/30 on 28/11/17. He began a precision medicine multimodal approach, making changes in his diet, exercise, mindfulness, and sleep, including a supplement program personalized to his laboratory abnormalities. He moved out of his moldy home and was treated for exposure to toxic mold as well as mercury.

After beginning his protocol, his MoCA score increased to 28/30. However, in 2019, he discontinued parts of the protocol for a few months, and noted a slight decline in his cognitive abilities, leading him to return for further evaluation and treatment. Reestablishing his original protocol mitigated his symptoms, and he once again said that he felt that his cognition was back to normal.

Follow-up at six years: He is currently employed, working as a private yacht captain, and reports not having any of the navigational problems or short-term memory deficits that he had been experiencing. His MoCA score was 30/30. His TGF-β1 improved from 3997→1614 pg/mL, his MMP-9 improved from 418→372 ng/mL, his homocysteine improved from 24.5→13.8 µmol/L, his vitamin B12 increased from 317→1200 pg/mL, vitamin D increased from 31→48 ng/mL, and zinc increased from 75→101 mcg/dL.

Comment: This man, homozygous for the ε4 allele of ApoE and with a strong family history of AD, displayed symptoms common in AD, such as difficulty with navigation and memory consolidation. He was found to have exposure to both mycotoxins and mercury. Detoxification, along with optimization of his nutrition, exercise, sleep, and stress level, led to improvements both symptomatically and according to cognitive testing. He underwent secondary decline when he discontinued parts of his personalized protocol, but responded to reestablishment of the protocol, and has remained without his initial complaints for six years since his presentation, including a continued ability to perform his job duties.

### 2.4. Patient 4

A 49-year-old woman noted progressive memory loss, which reached the point that she put a “sticky” note on the steering wheel of her car to remind her to drive on the right side of the road (as a jogger, she was used to staying on the left side, and she had begun to confuse the two). She took an on-line cognitive assessment, which placed her at the 35th percentile for her age, which concerned her because she had obtained an advanced degree following college and had always been superb in her test taking. Her husband, an airline pilot who flew overseas frequently, noted her further decline each time he was away for a week or more. Because of her relatively young age, she did not even consider the possibility of AD (despite a strong family history of dementia) until she took a genetic test (for other reasons), which revealed her to be homozygous for the ε4 allele of ApoE (ApoE 4/4). When she told her husband that she was concerned that she may have AD, he said, “That would explain a lot”.

She sought the advice of a neurologist, a recognized expert in the field of Alzheimer’s, who agreed that she did indeed have Alzheimer’s-related MCI. When she asked him if he could help her to avoid further decline, he replied, “Good luck with that”.

She began on a precision medicine protocol that addressed her insulin resistance and systemic inflammation, included a plant-rich mildly ketogenic diet (with blood beta-hydroxybutyrate levels typically in the 1.5–2.0 mM range), daily exercise, sleep hygiene, stress management, brain training, detoxification, and supplementation. Her cognitive testing improved from the 35th percentile to the 98th percentile.

Follow-up at 11 years: Her marked improvement has been sustained for 11 years, but she had two periods of re-decline: after five years of treatment, she noted a return of some of her earlier memory loss. The evaluation at that time revealed a very high TGF-β1 level (>40,000 pg/mL), and an infection with *Babesia*. She had had a tick bite 10 years earlier and had been treated successfully for Lyme disease after a characteristic bullseye rash appeared, but had not been treated for tick-borne coinfections. With treatment for *Babesia*, her cognition improved once again.

After 10 years, she again noted mild cognitive decline, and at that time, the evaluation revealed mycotoxin exposure. With treatment using the Shoemaker Protocol, she once again improved her cognition, and continues to score at >95th percentile on cognitive testing (most recent score on Elevate = 96.9th percentile for age). She is now 60 years of age and asymptomatic.

Comment: This patient, who is homozygous for the ε4 allele of ApoE (ApoE 4/4), responded very well to the initial treatment in which her insulin resistance was treated; she adopted a plant-rich, mildly ketogenic diet, she healed her gastrointestinal hyperpermeability, she exercised regularly, she adopted good sleep hygiene, managed her stress, and underwent brain training. However, she later suffered secondary decline, which responded well to the treatment of *Babesia* in one case and to the treatment of mycotoxins in the second case. She continues to be asymptomatic after 11 years, and continues to routinely score at >95th percentile on cognitive testing.

### 2.5. Patient 5

This 67-year-old government employee has been described previously [6], and at that time, she had only 2.5 years of follow-up. She has now been followed for over 11 years. She presented with progressive memory loss of a two-year duration. She held a demanding job that involved preparing analytical reports and extensive traveling, but found herself no longer able to analyze data or prepare the reports, and therefore considered quitting her job. When she read, by the time she reached the bottom of a page, she would have to start at the top once again, since she was unable to remember the material she had just read. She was no longer able to remember numbers, and had to write down even 4-digit numbers to remember them. She also began to have trouble navigating: even on familiar roads, she would become lost trying to figure out where to enter or exit the road. She also noticed that she would mix up the names of her pets, and forget where the light switches were in her home of many years.

Her mother had developed similar progressive cognitive decline in her early 60s, had become severely demented, entered a nursing home, and died at approximately 80 years of age. When the patient consulted her physician about her problems, she was told that she had the same problem her mother had had, and that there was nothing he could do about it. He wrote “memory problems” in her chart, and therefore the patient was turned down in her application for long-term care.

After being informed that she had the same problem as her mother had had, she recalled the many years of her mother’s decline in a nursing home. Knowing that there was still no effective treatment and subsequently losing the ability to purchase long-term care, she decided to commit suicide. She called a friend to commiserate, who referred her for evaluation.

She began the same precision medicine protocol utilized for the other patients in this report, and was able to adhere to some but not all of the protocol components. Nonetheless, after three months, she noted that her symptoms had abated: she was able to navigate without problems, remember telephone numbers without difficulty, prepare reports and do all of her work without difficulty, and read and retain information. She noted that her memory had become better than it had been in many years. On one occasion, she developed an acute viral illness, discontinued the program, and noticed a decline after 7–10 days, which reversed when she reinstated the program. Two and a half years later, at age 70, she remained largely asymptomatic and continued to work full time.

Follow-up at 11+ years: At 79 years of age, she continues to do well, works as a brain health coach, and recently completed a 100-mile bicycle ride in under 10 h.

Comment: This patient presented with memory and navigational complaints, and responded well to a personalized, multi-modal protocol. She had a brief period of secondary decline when she discontinued the protocol in association with a viral illness, but this reversed when she reinstated the protocol. She continues to do well, without cognitive complaints, at over 11 years after initiating treatment.

### 2.6. Patient 6

A 62-year-old man developed cognitive decline, which began appearing in 2014. He and his wife had noted that he was struggling to put his words together, prompting a workup at a university center of excellence, including a CT angiogram, all of which was negative. In January of 2016, his wife was startled when he forgot that he had purchased a tuxedo, and forgot where he had parked his car. He was an avid skier and had some altitude sickness as well. He underwent fluorodeoxyglucose PET scanning, which was indicative of AD, and a diagnosis of early onset AD (EOAD, since his symptoms had begun prior to 65 years of age), MCI stage, was made.

When his wife asked what could be done, she was told by the neurologist to “get his affairs in order, this is a bad disease that is always fatal”. He was started on memantine and given a follow-up appointment for 6 months. He was 64 years old at the time, and still working as an accountant. After the diagnosis, he felt hopeless and depressed.

He also felt frightened by all the cognitive symptoms he had been experiencing, and his wife brought him to one of the authors (MKR), who uses a precision medicine approach to treat cognitive decline. The patient had a full blood evaluation, volumetric MRI, cognitive testing, and was started on a precision medicine protocol. Initially this program proved to be very difficult for him, and he struggled most with the diet. He had been a long-time vegetarian and had enjoyed a great deal of pasta and foods with a higher carbohydrate content; in addition, it was discovered that he had been drinking a bottle of white wine nightly.

His evaluation revealed him to be ApoE 3/3, MTHFR C677T homozygous, with a homocysteine level of 9.3 micromolar. He had an elevated blood mercury level of 16.4 mcg/L. His morning cortisol level was elevated, and he gave a history of waking up at 3 AM nightly, ruminating anxiously. His MoCA score was 25/30, and his hippocampal volume was at the 13th percentile for his age.

He saw almost immediate improvement when he adhered to a diet without simple sugars and carbohydrates, and stopped drinking alcohol. He had another episode that resembled a stroke while skiing, and was advised by his physician to discontinue high-altitude visits. He continued to play tennis regularly, and worked on mindfulness and stress reduction.

Follow-up at 7 years: His MoCA scores improved from 25→28, and his hippocampal volume increased slightly, from the 13th percentile in 2018 to the 18th percentile in 2020. He has been on the program now for 7 years and has been able to sustain his improvements. When he deviates from the plant-rich, low-carbohydrate diet, however, he notes that it affects his cognition.

Comment: This patient had MCI due at least in part to AD pathophysiology, as revealed by his FDG-PET scan (his alcohol intake is likely to have played a role as well). He was given a very poor prognosis by his neurologist. However, a multi-modal approach, which predominantly involved nutrition and lifestyle for this patient, led to cognitive improvement that has been sustained for seven years. Given his current normal MoCA score and lack of symptoms, he no longer fits the criteria for MCI.

### 2.7. Patient 7

An 82-year-old, left-handed, retired female physician with a diagnosis of early AD was previously reported to have improved using a precision medicine approach [13].

She had noted mild cognitive problems for 20 years, which rapidly worsened in the year prior to being evaluated. She complained of poor recall of movies that she had watched and books that she had read. She had problems with name recall and was calling pets by the wrong names. She noticed that she was very susceptible to stress and fatigue. She described a great deal of stress when her son died a tragic death in 2001. Her partner stated that her memory was “abysmal”.

She has an active life, enjoying golfing, hiking, birding, and gardening. She worked in private practice as a psychiatrist until she retired at the age of 75. She has always been actively involved in civic associations and remains active in political circles.

She has a positive family history of dementia and memory problems: her mother developed dementia secondary to hydrocephalus, and her sister developed memory problems but died from an aneurysm at the age of 80.

A fluorodeoxyglucose-positron emission tomography (FDG-PET) scan revealed decreased glucose utilization in the anterior superior precuneus bilaterally and the anterolateral left temporal lobe, consistent with AD. She was diagnosed with early AD by her neurologist in 2016.

Her neurologist, after diagnosing AD, recommended treatment with donepezil and memantine. She refused both because she had read about their minimal effects on decline and because she had read about anecdotal successes with a precision medicine approach.

She began treatment with a precision medicine program in 2016, which included treatment for heavy metal toxicity. Her baseline brain training scores were in the 20th percentile for her age, and with continued training and adoption of her personalized protocol, these improved over approximately two years to the 94th percentile. Her symptoms improved markedly, although she did not become asymptomatic. Her partner, who had claimed that her memory was “abysmal”, noted that her memory had become “excellent”.

Follow-up at eight years: She has had sustained improvement over eight years with continued adherence to the programmatic approach. She had a temporary setback when she developed ehrlichiosis, but treatment with intravenous antibiotics led to improvement once again. She had a similar setback when a roof leak led to mycotoxin exposure, and detoxification led to improvement once again. Despite these repeated improvements, independence, excellent function, and repeatedly high scores on cognitive tests, a recent plasma p-tau 181 test was abnormal at 1.48 pg/mL (normal < 0.97 pg/mL).

Comment: This patient had mild cognitive impairment, and based on her FDG-PET scan results and plasma p-tau concentration, this was likely due to AD pathophysiology. She responded very well to a personalized, multi-modal protocol, with sustained improvement over seven years, punctuated by temporary setbacks associated with a tick-borne infection and mycotoxin exposure, both of which were successfully treated. Given her high scores on cognitive testing, high functioning, and minimal symptoms, her current diagnosis is subjective cognitive impairment (SCI) rather than MCI. However, her p-tau 181 level suggests that her underlying Alzheimer’s pathophysiology has not resolved completely.

A summary of the seven patients described above is included in Table 1.

## 3. Discussion

These case studies illustrate the point that some patients reverse their cognitive decline and achieve sustained improvement, in some cases for over a decade. We believe that this is the most important observation arising from the precision medicine approach because it suggests that the personalized protocol is indeed targeting the drivers of cognitive decline. This sustained improvement is in contrast to other treatments, such as donepezil or memantine, which have not yielded long-term benefits [1], and anti-amyloid antibodies, which lead to increased cerebral atrophy [2]. However, it is recognized that case studies cannot be compared to population data, which is why longer-term studies of the outcomes achieved with precision medicine protocols may be warranted. The current case reports are also in contrast to the natural history of patients with MCI, 10–20% of whom typically develop dementia each year [14], and who, in the longitudinal study by Krishnan et al. [15], declined an average of 1.7 points over 3.5 years. It should also be noted that the patients described here were declining at the time of treatment initiation and improved in temporal association with treatment, so it is likely that the sustained improvement was related to the treatment protocol.

These findings do not indicate how long the cognitive improvement in these patients may be sustained. Theoretically, however, it should be possible to sustain the improvement for the lifespan of each individual, since the hypothesized network insufficiency has been addressed, and thus there should be no further pro-Alzheimer’s signaling. However, longer-term follow-up will be required to confirm or refute this prediction.

In some cases, patients responded to treatment with improved cognition, but after success—in some cases for years—underwent a secondary decline or multiple periods of secondary decline. However, the decline was apparently driven by previously untreated factors, such as sleep apnea, chronic infections such as *Babesia*, toxins, or by a reduction in compliance, and when these factors were addressed, cognitive improvement occurred once again. This phenomenon of secondary decline, followed by secondary improvement, was noted previously, e.g., [6], and suggests that the protocol employed is addressing the drivers of cognitive decline. This notion is supported by the observation that reduced compliance may lead to cognitive decline, and the reintroduction of the protocol may once again lead to sustained improvement.

However, a limitation of this case report study is that it does not provide a denominator, so we do not report here what percentage of patients sustained their improvements. In a recent proof-of-concept trial, 84% of the patients with MCI or early dementia improved on this protocol, but we do not yet have long-term follow-up on those patients.

In the long term, it will be important to determine whether the addition of specific pharmaceuticals, such as cholinesterase inhibitors, beta-secretase inhibitors, or anti-amyloid antibodies, may enhance long-term outcomes. Meanwhile, this report suggests that a long-term clinical trial may be warranted in order to determine the frequency with which sustained improvements such as those reported here occur in patients with cognitive decline who are treated with a precision medicine protocol.

## 4. Conclusions

These results demonstrate the potential for long-term cognitive improvement in patients with AD, at least at the MCI stage; show that secondary decline may occur, and secondary improvement may then be achieved; and support the performance of a long-term cohort study.

## Figures and Tables

**Table 1:** Sustained cognitive improvement in patients treated with a precision medicine protocol.

	Symptoms	Diagnosis	Time Improved	Secondary Decline?	Secondary Improved?	Comments
75F	Paraphasic errors, memory	MCI (AD); E4+, PET+	7 years+	Yes (6 years)	Yes	OSA, crypto, mycotoxins
69M	Memory, prosop.	MCI (AD); E4+, PET+	10 years+	No	NA (not applicable)	No secondary decline
44M	Memory, navigation	MCI (AD); E4/4	6 years	Yes	Yes	Compliance
61F	Memory, prosop., navigation	MCI (AD); E4/4	11 years+	Yes × 2	Yes × 2	*Babesia*, mycotoxins
67F	Memory, navigation	MCI (AD)	11 years+	Yes × 3	Yes × 3	Compliance
71M	Memory, word finding	MCI (AD); PET+	7 years+	Yes	Yes	Compliance
82F	Memory	MCI (AD); PET+, pTau 181+	7 years+	Yes	Yes	*Ehrlichia*, *Borrelia*

## Data Availability

Data for this manuscript were obtained from patient records, and anonymized for publication. These data have not been deposited in a public data base.

## References

[1] Kennedy R.E., Cutter G.R., Fowler M.E., Schneider L.S. (2018). Association of Concomitant Use of Cholinesterase Inhibitors or Memantine with Cognitive Decline in Alzheimer Clinical Trials: A Meta-analysis. JAMA Netw. Open.

[2] Alves F., Kalinowski P., Ayton S. (2023). Accelerated Brain Volume Loss Caused by Anti–β-Amyloid Drugs: A systematic review and meta-analysis. Neurology.

[3] Bredesen D.E., Sharlin K., Jenkins K., Okuno M., Youngberg W., Hathaway A., Braud M., Henry P., Rusk I.N., Lawrence J. (2018). Reversal of Cognitive Decline: 100 Patients. J. Alzheimers Dis. Park..

[4] Toups K., Hathaway A., Gordon D., Chung H., Raji C., Boyd A., Hill B.D., Hausman-Cohen S., Attarha M., Chwa W.J. (2022). Precision Medicine Approach to Alzheimer’s Disease: Successful Pilot Project. J. Alzheimer’s Dis..

[5] Sandison H., Callan N.G.L., Rao R.V., Phipps J., Bradley R. (2023). Observed Improvement in Cognition During a Personalized Lifestyle Intervention in People with Cognitive Decline. J. Alzheimer’s Dis..

[6] Bredesen D.E. (2014). Reversal of cognitive decline: A novel therapeutic program. Aging.

[7] Bredesen D.E., John V. (2013). Next generation therapeutics for Alzheimer’s disease. EMBO Mol. Med..

[8] Bredesen D.E., Amos E.C., Canick J., Ackerley M., Raji C., Fiala M., Ahdidan J. (2016). Reversal of cognitive decline in Alzheimer’s disease. Aging.

[9] Rao R.V., Kumar S., Gregory J., Coward C., Okada S., Lipa W., Kelly L., Bredesen D.E. (2021). ReCODE: A Personalized, Targeted, Multi-Factorial Therapeutic Program for Reversal of Cognitive Decline. Biomedicines.

[10] Gualtieri C.T., Johnson L.G. (2006). Reliability and validity of a computerized neurocognitive test battery, CNS Vital Signs. Arch. Clin. Neuropsychol..

[11] Sabbagh M.N., Malek-Ahmadi M., Kataria R., Belden C.M., Connor D.J., Pearson C., Jacobson S., Davis K., Yaari R., Singh U. (2010). The Alzheimer’s questionnaire: A proof of concept study for a new informant-based dementia assessment. J. Alzheimer’s Dis..

[12] Bredesen D.E., Toups K., Hathaway A., Gordon D., Chung H., Raji C., Boyd A., Hill B.D., Hausman-Cohen S., Attarha M. (2023). Precision Medicine Approach to Alzheimer’s Disease: Rationale and Implications. J. Alzheimer’s Dis..

[13] Ross M.K., Raji C., Lokken K.L., Bredesen D.E., Roach J.C., Funk C.C., Price N., Rappaport N., Hood L., Heath J.R. (2021). Case Study: A Precision Medicine Approach to Multifactorial Dementia and Alzheimer’s Disease. J. Alzheimer’s Dis. Park..

[14] Alzheimers.gov What Is Mild Cognitive Impairment?. https://www.alzheimers.gov/alzheimers-dementias/mild-cognitive-impairment.

[15] Vos R., Boesten J., van den Akker M. (2022). Fifteen-year trajectories of multimorbidity and polypharmacy in Dutch primary care—A longitudinal analysis of age and sex patterns. PLoS ONE.

[16] Singer P.A. (2004). Consent to the publication of patient information. BMJ.

